# Investigation on the Association of Copper and Copper-to-Zinc-Ratio in Hair with Acute Coronary Syndrome Occurrence and Its Risk Factors

**DOI:** 10.3390/nu14194107

**Published:** 2022-10-03

**Authors:** Ewelina A. Dziedzic, Agnieszka Tuzimek, Jakub S. Gąsior, Justyna Paleczny, Adam Junka, Mirosław Kwaśny, Marek Dąbrowski, Piotr Jankowski

**Affiliations:** 1 Medical Faculty, Lazarski University in Warsaw, 02-662 Warsaw, Poland; 2 Department of Internal Medicine and Geriatric Cardiology, Centre of Postgraduate Medical Education, 01-813 Warsaw, Poland; 3 Department of Pediatric Cardiology and General Pediatrics, Medical University of Warsaw, 02-091 Warsaw, Poland; 4 Department of Pharmaceutical Microbiology and Parasitology, Faculty of Pharmacy, Wroclaw Medical University, 50-556 Wroclaw, Poland; 5 Institute of Optoelectronics, Military University of Technology, 00-908 Warsaw, Poland; 6 Department of Cardiology, Bielanski Hospital, 01-809 Warsaw, Poland; 7 Department of Epidemiology and Health Promotion, School of Public Health, Centre of Postgraduate Medical Education, 01-826 Warszawa, Poland

**Keywords:** copper (Cu), copper-to-zinc-ratio (Cu/Zn-ratio), coronary artery disease (CAD), acute coronary syndrome (ACS)

## Abstract

The prevalence of coronary artery disease (CAD) increases every year; however, the impact of microelements on its underlying cause—atherosclerosis—is still unclear. Copper plays numerous regulatory roles in cardiovascular health and was suggested to influence the classic risk factors for CAD. The copper-to-zinc-ratio (Cu/Zn-ratio) reflects systemic oxidative stress–one of the factors in the complex pathogenesis of atherosclerosis. Hair incorporates metal ions during its growth; thus, it reflects the metal exposure that occurred over the last 4–8 weeks. The aim of the presented study was to verify the association between Cu and Cu/Zn-ratio content and the occurrence of acute coronary syndrome (ACS) in the hair of 133 patients who underwent coronary angiography due to suspected ACS. Additionally, association between Cu and Cu/Zn-ratio and selected risk factors for CAD was analyzed. Neither Cu nor Cu/Zn-ratio levels were associated with the occurrence of ACS, regardless of its type (UA/NSTEMI/STEMI). We did not find a significant association between Cu content in hair and risk factors for CAD. The Cu/Zn-ratio in hair was significantly correlated only with body mass index. The relationship of Cu content and Cu/Zn-ratio in hair with CAD, its risk factors and ACS appears to be complex and requires further well-designed research.

## 1. Introduction

Coronary artery disease (CAD) is a growing threat to global health in both developing and developed countries, with an increasing prevalence and morbidity every year [1]. By 2060, 28.7 million patients will be diagnosed with CAD, 12.9 million with heart failure, and 16.0 million with myocardial infarction in the United States alone [2]. CAD is a clinical manifestation of atherosclerosis—a systemic inflammatory process in the walls of the arteries combined with lipid accumulation in the matrix of the intima, which progresses slowly; thus, the effects of this disease are seen mainly in the elderly [3].

Copper plays a crucial role in cardiovascular health. It is involved in cardiomyocyte metabolism [4], and as a cofactor of various enzymes, such as Cu/Zn superoxide dismutase, lysyl oxidase, and ceruloplasmin, it indirectly influences the strength and integrity of blood vessels by reducing oxidative stress and the regulation of peripheral blood flow [5]. Its deficiency negatively influences the main risk factors for CAD, causing increased cholesterol levels [6], dyslipidemia [7], increased inflammatory response [8], increased blood pressure [9,10], high hemoglobin glycation [11], and increased body mass index (BMI) [10,12]. Interestingly, elevated serum copper can lead to increased production of reactive oxygen species and oxidative stress [13,14]. This results in oxidation of lipids [15], including LDL, which play a key role in atherosclerosis [16,17]. Higher levels of copper were associated with the diagnosis of ischemic cardiomyopathy [17], an increase in the ten-year coronary risk [10], and an increased risk of mortality from all causes, as well as cardiovascular causes [18]. Therefore, both Cu deficiency and excess can lead to progression of atherosclerosis and CAD [19,20,21,22]. The copper-to-zinc-ratio (Cu/Zn-ratio) has been reported to reflect systemic oxidative stress [23], and predict all-cause mortality in elderly people over 70 years of age [24]. Cu/Zn-ratio may also influence some risk factors for CVD—hypercholesterolemia [25] and type 2 diabetes mellitus [26,27]. In diabetic patients, the serum Cu/Zn-ratio was inversely correlated with serum total cholesterol [28]. Furthermore, this indicator may be influenced by low-grade systemic inflammation [29,30].

Cu absorption differs due to various factors, including age, sex, bioavailability of other microelements, and drug intake; therefore, serum levels of copper may not be an accurate estimate of its dietary intake, nor its total status in the body [31]. Therefore, other techniques, such as hair analysis, which provide exposure measurement over 4–8 weeks [32], seem to be worth exploring [33]. Hair is a metabolic end product, which incorporates metals into its structure during growth. It averages variations in blood and urine measurements, which can vary hourly and provide exposure assessment over a short period only [34,35]. Hair samples are stable and safer to handle than blood or urine. Furthermore, they are non-invasive to obtain and their analysis may be easily acceptable as a future screening tool [36]. Inductively coupled plasma-optical emission spectrometry (ICP-OES) uses argon plasma heat to excite atoms in samples. During deexcitation, the atoms emit light, which the spectrum allows for the determination of the exact elemental composition of the sample [37].

Taking into account inconclusive data on the influence of Cu on the cardiovascular system, the main objective of this research was to analyze the association between Cu content in hair and the occurrence of acute coronary syndrome (ACS) in patients with coronary artery disease (stable CAD vs. ACS subgroups). In a recent study, we did not find a significant correlation between the Zn content in the hair and the occurrence of ACS [38]. Therefore, the Cu/Zn-ratio in hair, which describes the balance between these two elements, was analyzed in a similar way to the Cu content. Furthermore, the correlation between the aforementioned factors in hair and selected known risk factors for CAD was also investigated. This research paper and the study on the Zn content in hair and CAD [38] are parts of the project on the relationship between the content of microelements in hair and various aspects of this disease.

## 2. Materials and Methods

### 2.1. Study Population

The final data set used in this investigation consisted of 133 patient cases (37 females), who underwent coronary angiography due to suspected ACS in outpatient care between 2013 and 2017 at Bielanski Hospital, Department of Cardiology (Warsaw, Poland). The same cohort was described elsewhere [38]. All patients were residents of Warsaw with no history of occupational exposure to chemical elements. Each patient agreed to participate in the study by filling out a written informed consent form. Exclusion criteria included: dying or permanently waving hair in a 3 cm segment counting from the scalp, significantly elevated inflammatory markers, active neoplastic diseases or paraneoplastic syndromes, viral or bacterial infection, chronic kidney disease (stages III–V), ingesting medications or dietary supplements containing copper or zinc, or using shampoos with increased content of those elements. The study was carried out according to the principles of the Declaration of Helsinki and was approved by the Medical University of Warsaw bioethics committee.

### 2.2. Sample Collection and Analysis

Hair samples were collected from a few separate scalp sites in the back of the head, close to the skin. The samples obtained, weighing between 0.2 and 0.3 g, were washed with a non-ionic detergent water solution of (Triton X-100, Sigma Aldrich, Poland) in a 1:100 dilution and ultrasonic bath for 5 min, then sequentially rinsed with high purity water, acetone, water, and then dried. Solid hair samples, 0.15 g each, were dissolved in a closed polypropylene vial (8 mL) using a mixture of 4 mL of 65% nitric acid (Merck, Darmstadt, Germany) and 1 mL of 30% hydrogen peroxide (Merck, Darmstadt, Germany) followed by incubation at 80 °C for 30 min in a microwave station. The samples were then cooled to room temperature and diluted to a final volume of 10 mL with Milli-Q water and analyzed with a previously validated method using an ICP-OES spectrometer (iCAP7400, Thermo Scientific, Waltham, MA, USA) [39]. The concentrations of Cu and Zn in the obtained solutions, representing a total content in the hair samples, were calculated based on results obtained for certified standards: CGZN1 and CGCU1 (Inorganic Ventures, Christiansburg, VA, USA) for Zn and Cu, respectively. Reference ranges for Cu or Cu/Zn-ratio were not found in the literature. The PubMed/MEDLINE database was searched using combinations of keywords: “hair” and at least one of the following: “obesity”, “overweight”, “BMI”, “hypertension”, “infarction”, “lipid”, “diabetes”, “ischemic”, “coronary”, “angina” and at least one of the following: “cu”, “copper”, “ratio”, “element”, “microelement”, “metal” to find articles on Cu content or Cu/Zn-ratio in hair and its correlation with ACS occurrence or known risk factors. A total of 1525 articles were found, 10 of which were considered to be relevant to this research. We excluded two articles due to the use of non-standard units that were not compatible with the rest of the summary. Additionally, three publications on Cu content in hair of healthy adults or the elderly are presented for a baseline. The results presented in these research papers are collected in Table S1.

### 2.3. Laboratory and Clinical Data

Anthropometric and laboratory data were collected from patient electronic files. BMI was determined as the ratio of weight (kg) to the square of height (m^2^) to diagnose obesity or overweight based on WHO criteria [40]. Blood samples for laboratory tests, including serum levels of total cholesterol (TC), high-density lipoprotein cholesterol (HDL), and triglycerides (TG) were collected on the day of admission by cephalic venepuncture and examined in the hospital laboratory using standard techniques. Low-density lipoprotein cholesterol (LDL) was calculated from Friedewald formula. Hyperlipidemia was diagnosed in patients whose lipid profile did not meet the treatment goals for their respective risk level according to the 2019 ESC/EAS Guidelines for the management of dyslipidemias [41]. Diabetes mellitus was diagnosed if laboratory examination revealed fasting blood glucose levels exceeding ≥7.0 mmol/L (126 mg/dL) twice, or random blood glucose levels exceeding ≥11.1 mmol/L (200 mg/dL) accompanied by hyperglycemia, or blood glucose at 120 min during an oral glucose tolerance test exceeding ≥11.1 mmol/L (200 mg/dL) [42]. Hypertension (HTN) was defined as blood pressure exceeding 140/90 mmHg during in-office measurement, as described in the 2021 European Society of Hypertension Practice Guidelines [43].

### 2.4. Coronary Angiography

According to the 2021 American College of Cardiology/American Heart Association/Society for Cardiovascular Angiography and Interventions Guideline for Coronary Artery Revascularization, the leading imaging method of stenosis in CAD and its complication, ACS, is coronary angiography [44]. It uses X-rays and iodinated contrast injected through radial or femoral artery access to reveal coronary artery stenosis. The presence of ≥1 stenosis with ≥50% diameter in at least one major coronary artery was considered significant. Uncertainties regarding the percentage of stenosis were solved by measuring the fractional flow reserve. Acute coronary syndrome (ACS), unstable angina (UA), non-ST segment elevation myocardial infarction (NSTEMI), and ST segment elevation myocardial infarction (STEMI) were diagnosed according to criteria of the European Society of Cardiology. ACS was defined as an increase in the concentration of markers of myocardial injury with the coexistence of at least one of the following: symptoms of stenocardia, changes in the ECG suggestive of ischemia, results of imaging tests showing myocardial necrosis or coronary artery thrombus identified on coronary angiography. UA was described as myocardial ischemia at rest or with minimal exertion in the absence of acute cardiomyocyte injury. STEMI and NSTEMI were defined as ACS with or without persistent ST-segment elevation (>20 min), respectively [45].

### 2.5. Statistical Analysis

Normality of data was assessed using Kolmogorov–Smirnov test. To identify associations between dichotomous and categorical data, the chi-square statistic was used. The Mann–Whitney test and the Kruskal–Wallis analysis by rank were used to compare the values between two groups and determine the dependence between more than two groups of patients, respectively. To evaluate the relationship between the variables, the R Spearman correlation test was used. Multivariable regression analysis was carried out to identify independent determinants of Cu and Cu/Zn-ratio—variables with a skewed distribution were logarithmically transformed (ln). A *p*-value < 0.05 was regarded as statistically significant. Statistical analysis were conducted using Statistica 13 (StatSoft Inc., Tulsa, OK, USA). GraphPad Prism 5 (GraphPad Software Inc., San Diego, CA, USA, 2005) was used to create figures.

## 3. Results

### 3.1. Population Characteristics

**Figure 1:**
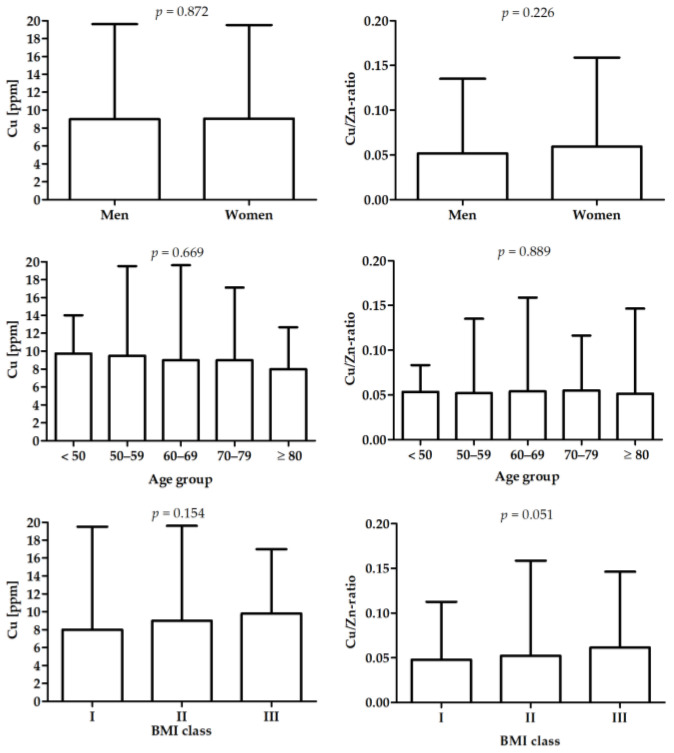
Differences in Cu and Cu/Zn-ratio between male and female, age groups, and BMI groups.

Details on the study group are presented in Table 1 and in our previous study [38]. Differences in Cu and Cu/Zn-ratio between male and female patients, age, and BMI subgroups are presented in Figure 1.

### 3.2. Differences in Cu and Cu/Zn-Ratio Levels between Patients with ACS and Stable CAD

Table 2 presents the results for patients with different diagnosis: stable CAD, STEMI, NSTEMI, UA in measured parameters.

There was a significant difference in distribution of patients with smoking status. Patients with STEMI presented the highest value of LDL (*p* = 0.018 vs. patients with stable CAD). There were no significant differences in Cu and Cu/Zn-ratio between patients with different diagnoses.

### 3.3. Association between Cu, Cu/Zn-Ratio, and Selected Parameters

**Figure 2:**
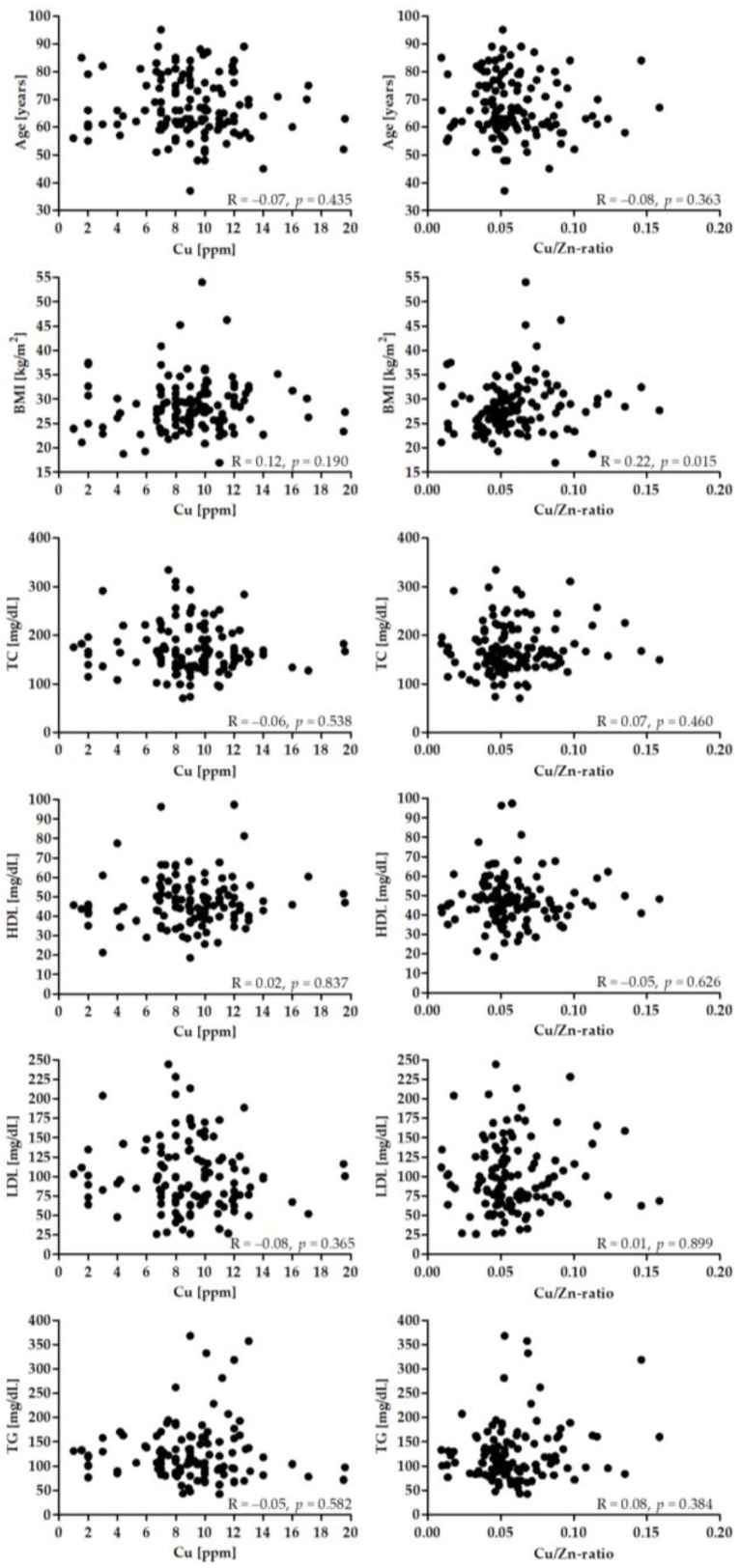
Correlation between Cu (left panel), Cu/Zn-ratio and age, BMI and lipid profile.

Figure 2 presents the correlation between the Cu and Cu/Zn-ratio and age, BMI, and lipid profile. There was a significant correlation between BMI and Cu/Zn-ratio (*p* < 0.05).

**Figure 3:**
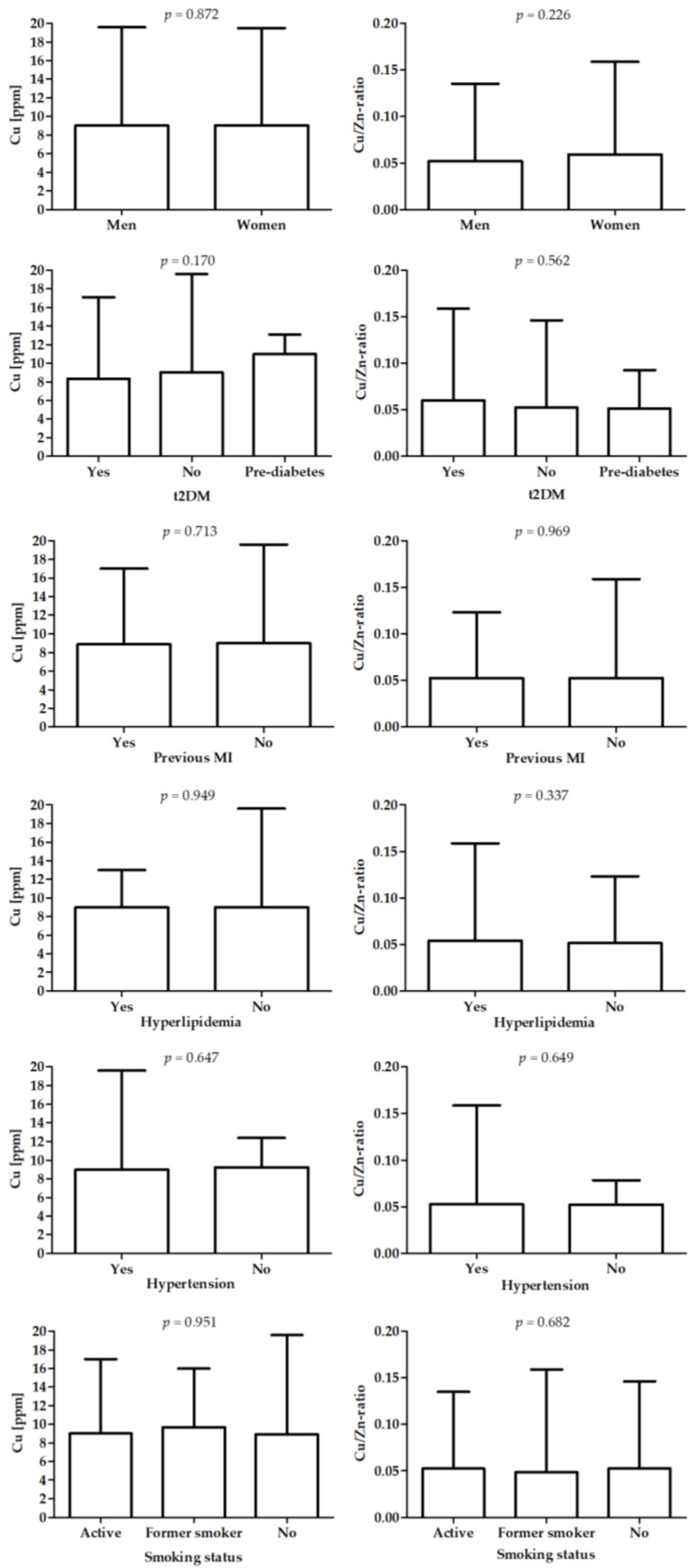
Differences in Cu (left panel) and Cu/Zn-ratio (right panel) between patients with different diagnoses.

Figure 3 presents differences in Cu and Cu/Zn-ratio between patients with different diagnoses.

The results of multivariable regression analysis are presented in Table 3 for logarithmically transformed Cu and in Table 4 for logarithmically transformed Cu/Zn-ratio. Both models were not statistically significant (F = 0.905, *p* = 0.556 for Cu and F = 0.479, *p* = 0.930 for Cu/Zn-ratio) and accounted for 13% (R^2^ = 0.125) and 7% (R^2^ = 0.071) of the microelements’ variance, respectively.

## 4. Discussion

Copper is involved in multiple metabolic processes and its serum concentration was previously correlated with CAD, its risk factors, and the occurrence of its complication—ACS. However, data on the relationship between hair element content and these issues are limited and often remain ambiguous. Therefore, this study aimed to analyze not only the association between Cu content and Cu/Zn-ratio in hair and the risk factors for CAD, but also the correlation between those elements in hair and the occurrence of acute coronary syndrome (ACS) in patients with coronary artery disease (stable CAD vs. ACS subgroups). No relation was found between hair Cu levels and classic risk factors for CAD. Furthermore, the Cu content and Cu/Zn-ratio in hair did not differentiate patients with ACS or stable CAD independently of a history of previous ACS or the subtype of ACS (UA vs. STEMI vs. NSTEMI). However, we found a positive association of Cu/Zn-ratio with BMI, but not with other risk factors.

The relationship between BMI, Cu, and Cu/Zn-ratio was previously studied [46,47,48,49,50]. A positive relation of BMI to Cu in serum was first reported in 32 obese patients compared to the healthy group in 2001 [46], followed by similar results in hair samples of nearly 400 Taiwanese women [47]. These data have also shown the correlation of the Cu/Zn-ratio in hair with BMI, similarly to another Saudi Arabian study on diabetic and obese women [48]. A Korean study has shown a relationship between Cu and Cu/Zn-ratio content in hair and neutrophil-to-lymphocyte-ratio (NLR) in 56 patients with BMI greater than 23 kg/m2 [49]. NLR is an important marker of chronic inflammation [50] and a significant indicator of inflammation in cardiovascular disease [51,52,53]. Therefore, the Cu/Zn-ratio would reflect the increased burden of inflammation oxidation, as a high Cu level increases the oxidative damage to lipids and proteins, while decreased levels of anti-inflammatory Zn could not counteract the influence of Cu [49]. However, two other articles found that Cu and Zn hair contents were not correlated with obesity compared to subjects of normal weight [54,55]. These discrepancies in the results may be caused by the diverse diets of patients from different datasets, as each of these studies appear to be limited to a single cultural background of subjects.

There are very limited data on the association of copper with other risk factors for CAD: hypertension, diabetes, hypercholesterolemia, and smoking, especially in the context of their concomitance with CAD. A study carried out on hair samples obtained from nearly 400 HTN patients revealed lower levels of Cu and Zn in the hair compared to a healthy control [56]. These results do not corroborate the data obtained by us and Vivoli et al., who found no difference between patients with or without hypertension [57]. Another risk factor for CAD, diabetes, was previously found to be not correlated with Cu values in hair compared to healthy individuals [58], which is consistent with our results. On the other hand, a Japanese study in more than 100 patients has shown that the Cu content in hair decreases with increasing glycated hemoglobin, which was suggested to be caused by diabetic nephropathy [59]. Previously, similar results were obtained in another article on patients diagnosed with diabetes mellitus, as well as descendants of both parents who were diagnosed with diabetes. This could suggest that the elemental imbalance occurs before the diagnosis of diabetes has been made [60], but the data on Cu tissue distribution in diabetic patients were previously pointed out as controversial [60,61]. Interestingly, a previously mentioned study in obese and diabetic women revealed that not only Cu/Zn-ratio was higher in obese patients, but it could also differentiate between diabetic and non-diabetic patients [48]. However, similarly to our study, a research on another group of obese diabetic patients found no correlation between Cu values and Cu/Zn-ratio in hair [28]. Furthermore, their data have not found a correlation between total cholesterol and triacylglycerol concentrations and Cu content or Cu/Zn-ratio in hair, neither in diabetic patients nor in healthy individuals. These results are consistent with a comparable study in hypertensive and obese patients with insulin resistance [62]. Smoking, another risk factor for CAD, had no influence on Cu and Zn in hair in patients with CAD [63,64]. In contrast, data from a similar study on patients with myocardial infarction (MI) revealed that they had lower Cu and Zn in hair compared to a healthy control [65]. The relation between Cu content and Cu/Zn-ratio in hair samples and risk factors for CAD remains unclear, as the results shown above often remain ambiguous.

In our group of patients, the Cu content and Cu/Zn-ratio in hair were not found to differentiate patients with ACS or stable CAD independently of a history of previous ACS or the subtype of ACS (UA vs. STEMI vs. NSTEMI). The lack of difference may be the result of the character of the analyzed groups (CAD vs. ACS). These data are difficult to compare with other research, as the majority of studies were based on the comparison of patients with CAD or ACS (earlier called MI) to healthy control. A Pakistani study in 82 patients with CAD compared to a healthy control revealed a higher Cu content in the hair and lower levels of zinc in the diagnosed group. Furthermore, patients with stable CAD had relatively higher average Cu levels compared to those with UA [63]. On the contrary, a Pakistani study comparing the Cu and Zn content in hair two years later did not find a significant difference between patients with and without CAD [64]. Similarly, Chinese research on hair from 46 elderly patients with HTN and CAD did not find a significant correlation of hair Cu content compared to the control group, but the Cu/Zn-ratio was significantly lower in diagnosed patients [66]. Another Chinese study in 24 patients with CAD found that Cu and Zn levels in the hair were higher than in a healthy control, but without stating the *p*-value [67]. Data on the relation of Cu content with MI were previously summarized in the 2015 meta-analysis that compared the hair and serum Cu content in patients with and without a history of MI. It revealed that despite finding the correlation in serum of the entire dataset of 18 studies, it has high heterogeneity between subsets. This led to an analysis of ethnic subgroups, which revealed that a significant difference in serum Cu was found in Asians, but not in Caucasians. Due to limited data on Cu content in hair, the analysis of geographic location subgroups was performed and a statistically significant difference was found between patients with and without a history of MI in Pakistan, but not in India [68]. A Pakistani study in 2015 confirmed the previous results that Cu content in hair is higher in MI patients compared to the healthy control [65]. There are also conflicting data on the correlation between Cu/Zn-ratio in hair of patients with MI. A study on hair samples of 29 male MI survivors revealed that the Cu/Zn-ratio was significantly lower compared to the control, but the Zn content was higher [69]. In contrast, a study in a larger group of 193 patients with a history of MI (104 men and 89 women) resulted in the finding of an opposing correlation of a higher Cu/Zn-ratio in hair, but lower levels of Zn compared to healthy subjects [70]. These interesting facts should initiate further research which would take into consideration a wide variety of factors, such as ethnicity, diet, and environment, as these factors may have greater influence than it is thought. Furthermore, this would allow a better understanding of the relationship of the Cu content and the Cu/Zn-ratio in hair and the occurrence of CAD and MI in different groups of patients.

This study has a few limitations that should be recognized. Despite the considerable number of patients involved, this investigation was retrospective, cross-sectional, and observational. Thus, the exact cause-and-effect relationship between the Cu/Zn-ratio in hair and CAD remains unclear. Although relatively wide exclusion criteria were defined, the inability to isolate various impacts on hair Cu and Cu/Zn-ratio content remains due to probable different diet patterns, bioavailability of microelements in food, drug intake and other inflammation causes other than heart disease not being diagnosed at the time of the study being conducted. Reference values for the Cu and Zn content as well as Cu/Zn-ratio are also yet to be established. Therefore, Table S1 compares our results with data from selected articles referenced in our manuscript, along with two others [70,71] included in the meta-analysis mentioned above [68], as well as articles aimed at assessing possible reference values [72,73,74].

All the data mentioned above, including the results of this study, suggest that the relationship of Cu and Cu/Zn-ratio content in hair with CAD, its risk factors, and MI appears to be much more complex than expected. Taking into account the growing burden of cardiovascular diseases and the possible advantages of hair sample usage, this topic requires further well-designed and thorough research in diverse populations to be considered applicable in future clinical practice.

## 5. Conclusions

This study depicts a correlation of BMI with the copper-to-zinc-ratio in the hair of patients with CAD diagnosed with coronary angiography. The Cu content in the hair of patients with stable CAD was not significantly different compared to patients with ACS. The relationship of hair Cu and Cu/Zn-ratio content with CAD, its risk factors, and MI appears to be complex and needs further well-designed research.

## Figures and Tables

**Table 1:** Participants’ characteristics.

Variable	Values
Age (years)	65 (60–75)
BMI (kg/m^2^)	28 (25–31)
Diabetes mellitus (yes/pre-diabetes/no)	42 (32%)/7 (5%)/84 (63%)
TC (mg/dL)	166 (137–204)
HDL (mg/dL)	46 (40–54)
LDL (mg/dL)	91 (70–126)
TG (mg/dL)	111 (88–154)
Hyperlipidemia (yes/no)	55 (41%)/68 (51%)
Hypertension (yes/no)	114 (86%)/19 (14%)
Smoking (active/former smoker/no)	40 (30%)/17 (13%)/76 (57%)
Stable CAD/ACS	66 (50%)/67 (50%)
Previous MI (yes/no)	40 (30%)/93 (70%)
Cu (ppm)	9 (7–11)
Cu/Zn-ratio	0.05 (0.04–0.07)

Data presented as number (%) or median (interquartile range: 25th–75th percentiles). BMI—body mass index; TC—total cholesterol; HDL—high-density lipoprotein cholesterol; LDL—low-density lipoprotein cholesterol; TG—triglycerides; MI—myocardial infarction.

**Table 2:** Differences in selected parameters between patients with different diagnoses.

Variable	Stable CAD	STEMI	NSTEMI	UA	*p*-Value
Number (♀/♂)	21 (16%)/45 (34%)	7 (5%)/25 (19%)	5 (4%)/15 (11%)	4 (3%)/11 (8%)	0.758
Age (years)	63 (61–74)	62 (57–80)	74 (62–79)	66 (64–77)	0.193
BMI (kg/m^2^)	28 (25–32)	28 (25–30)	27 (25–33)	29 (28–30)	0.632
Diabetes mellitus (yes/pre-diabetes/no)	23 (17%)/3 (2%)/40 (30%)	9 (7%)/2 (2%)/21 (16%)	4 (3%)/2 (2%)/14 (11%)	6 (5%)/0/9 (7%)	0.735
TC (mg/dL)	160 (135–191)	170 (155–221)	161 (133–190)	167 (132–211)	0.174
HDL (mg/dL)	46 (41–58)	47 (38–50)	44 (37–48)	45 (43–53)	0.505
LDL (mg/dL)	79 (65–118)	106 (85–153)	100 (74–130)	102 (76–139)	0.028
TG (mg/dL)	125 (94–162)	105 (88–123)	97 (70–134)	101 (85–146)	0.043
Hyperlipidemia (yes/no)	29 (24%)/33 (27%)	13 (11%)/17 (14%)	6 (5%)/11 (9%)	7 (6%)/7 (6%)	0.826
Hypertension (yes/no)	55 (41%)/11 (8%)	25 (19%)/7 (5%)	19 (14%)/1 (1%)	15 (11%)/0	0.126
Smoking (active/former smoker/no)	18 (14%)/15 (11%)/33 (25%)	13 (10%)/1 (1%)/18 (14%)	6 (5%)/0/14 (11%)	3 (2%)/1 (1%)/11 (8%)	0.028
Previous MI (yes/no)	22 (17%)/44 (33%)	7 (5%)/25 (19%)	6 (5%)/14 (11%)	5 (4%)/10 (8%)	0.698
Cu (ppm)	9 (7–11)	9 (7–10)	8 (7–10)	10 (8–11)	0.444
Cu/Zn-ratio	0.05 (0.04–0.08)	0.05 (0.05–0.06)	0.05 (0.04–0.06)	0.06 (0.05–0.07)	0.209

Data presented as number (%) or median (interquartile range: 25th–75th percentiles). BMI—body mass index; TC—total cholesterol; HDL—high-density lipoprotein cholesterol; LDL—low-density lipoprotein cholesterol; TG—triglycerides; MI–myocardial infarction.

**Table 3:** Multivariable regression analysis for Cu.

Determinant	Β (SE β)	*p*	Partial Correlation	Tolerance	R^2^
Age (years)	−0.12 (0.12)	0.354	−0.10	0.70	0.30
Sex (♀/♂)	0.05 (0.12)	0.672	0.05	0.77	0.23
BMI (kg/m^2^)	−0.02 (0.11)	0.871	−0.02	0.86	0.14
Diagnosis (stable CAD/ACS)	0.17 (0.12)	0.156	0.16	0.74	0.26
TC (mg/dL)	0.60 (0.69)	0.385	0.10	0.02	0.98
HDL (mg/dL)	0.06 (0.22)	0.796	0.03	0.22	0.78
LDL (mg/dL)	−0.64 (0.57)	0.266	−0.12	0.03	0.97
TG (mg/dL)	−0.03 (0.21)	0.886	−0.02	0.25	0.75
Hyperlipidemia (no/yes)	0.06 (0.16)	0.687	0.04	0.41	0.59
Hypertension (no/yes)	0.06 (0.11)	0.586	0.06	0.84	0.16
Diabetes mellitus (no/yes)	−0.17 (0.12)	0.144	−0.16	0.80	0.20
Previous MI (no/yes)	−0.08 (0.11)	0.483	−0.08	0.89	0.11
Smoking (no/yes)	−0.12 (0.12)	0.301	−0.11	0.79	0.21

BMI—body mass index; CAD—coronary artery disease; ACS—acute coronary syndrome; TC—total cholesterol; HDL—high-density lipoprotein cholesterol; LDL—low-density lipoprotein cholesterol; TG—triglycerides; MI—myocardial infarction.

**Table 4:** Multivariable regression analysis for Cu/Zn-ratio.

Determinant	Β (SE β)	*p*	Partial Correlation	Tolerance	R^2^
Age (years)	−0.08 (0.13)	0.511	−0.07	0.70	0.30
Sex (♀/♂)	−0.01 (0.12)	0.908	−0.01	0.77	0.23
BMI (kg/m^2^)	−0.01 (0.12)	0.987	−0.01	0.86	0.14
Diagnosis (stable CAD/ACS)	0.19 (0.12)	0.124	0.17	0.74	0.26
TC (mg/dL)	0.34 (0.71)	0.634	0.05	0.02	0.98
HDL (mg/dL)	0.05 (0.23)	0.833	0.02	0.22	0.78
LDL (mg/dL)	−0.38 (0.59)	0.516	−0.07	0.03	0.97
TG (mg/dL)	0.10 (0.21)	0.645	0.05	0.25	0.75
Hyperlipidemia (no/yes)	0.07 (0.17)	0.660	0.05	0.41	0.59
Hypertension (no/yes)	0.05 (0.12)	0.668	0.05	0.84	0.16
Diabetes mellitus (no/yes)	0.04 (0.12)	0.730	0.04	0.80	0.20
Previous MI (no/yes)	0.01 (0.11)	0.908	0.01	0.89	0.11
Smoking (no/yes)	−0.01 (0.12)	0.954	−0.01	0.79	0.21

BMI—body mass index; CAD—coronary artery disease; ACS—acute coronary syndrome; TC—total cholesterol; HDL—high-density lipoprotein cholesterol; LDL—low-density lipoprotein cholesterol; TG—triglycerides; MI—myocardial infarction.

## Data Availability

Data can be provided by the corresponding author upon reasonable request.

## Supplementary Materials

The following are available online at https://www.mdpi.com/article/10.3390/nu14194107/s1, Table S1: Comparison of literature data on Cu, Zn content, and Cu/Zn-ratio in hair.
