# Supplementary material for: Investigation on the Association of Copper and Copper-to-Zinc-Ratio in Hair with Acute Coronary Syndrome Occurrence and Its Risk Factors
Source: Nutrients. 2022 Oct 3;14(19):4107. doi: 10.3390/nu14194107 (PMC9571797; doi:10.3390/nu14194107)
Supplement: Supplementary file 1 [file nutrients-14-04107-s001.zip › Supplementary Material.pdf]

Table S1. Comparison of literature data on Cu, Zn content, and Cu/Zn-ratio in hair.

Cohort	Cu content		Zn content		Cu/Zn-ratio	
	**Control**	**Diagnosed**	**Control**	**Diagnosed**	**Control**	**Diagnosed**
Data presented in our study – CAD, (ppm)	NA	9.0 (1.0 – 19.6)	NA	ACS 166.0 (39.0–285.0)<br>CAD 166.5 (25.0–495.0)<br>[38]	NA	0.053 (0.009 – 0.159)
Obesity, (µg/g) [47]	10.2 ± 7.1 (38.2–3.2)	BMI 26-35: 15.2 ± 10. (65.2–7.5)<br>BMI>35: 16.2 ± 10.8 (66.5–6.5)	216.0 ± 121.7 (675.4–86.3)	BMI 26-35: 181.3 ± 91.2 (489.0–36.5)<br>BMI>35: 176.6 ± 83.9 (385.5–100.3)	0.05*	BMI 26-35: 0.084*<br>BMI>35: 0.091*
Overweight or obesity, (mg/100 g) [49]	NA	4.8 ± 7.2	NA	18.4 ± 10.1	NA	0.3 ± 0.6
Obesity, (µg/g) [54]	14.5 (10.7–25.9)	13.5 (12.0–18.6)	198.7 (160.3–227.5)	160 (132–188)	NA	NA
CAD, (µg/g) [63]	12.5 (2.9–36.3)	16.8 (4.5–30.6)	260.4 (66.7–1240)	212.1 (46.5–490.7)	NA	NA
Hypertension, (µg/g) [66]	6.5 ± 0.3	6.7 ± 0.7	118.6 ± 25.5	107.2 ± 21.8	0.055 ± 0.012*	0.065 ± 0.013*
ACS, (ppm) [69]	8.5 ± 4.1	7.4 ± 2.2	133 ± 34	158 ± 51	0.061 ± 0.017*	0.046 ± 0.011*
ACS, (µg/g) [70]	♂ 11.5 ± 3.7†<br>7.3 ± 4.2‡<br>5.2 ± 3.81¶	♂ 17.5 ± 6.2†<br>13.3 ± 7.1‡<br>9.2 ± 6.8¶	♂ 206.1 ± 14.0†<br>188.0 ± 15.8‡<br>154.4 ± 16.9¶	♂ 169.2 ± 18.2†<br>149.4 ± 15.7‡<br>107.7 ± 17.1¶	♂ 0.057†*<br>0.04‡*<br>0.035¶*	♂ 0.106†*<br>0.091‡*<br>0.088¶*
	♀ 11.9 ± 4.4†<br>8.2 ± 5.7‡<br>6.1 ± 4.9¶	♀18.2 ± 5.5†<br>15.5 ± 6.6‡<br>9.6 ± 7.3¶	♀209.2 ± 8.3†<br>189.0 ± 14.6‡<br>157.2 ± 18.1¶	♀181.1 ± 14.3†<br>167.1 ± 24.6‡<br>120.1 ± 33.0¶	♀ 0.059†*<br>0.044‡*<br>0.040¶*	♀ 0.104†*<br>0.095‡*<br>0.083¶*
ACS, (µg/g) [71]	♂ 11.5 ± 2.9	♂ 13.9 ± 2.7[1]<br>13.6 ± 2.1[2]<br>12.4 ± 1.9[3]	♂212.1 ± 16.5	♂162.4 ± 14.5[1]<br>145.9 ± 10.7[2]<br>120.0 ± 11.1[3]	NA	NA
	♀ 11.9 ± 3.6	♀14.2 ± 2.5[1]<br>13.2 ± 2.4[2]<br>12.7 ± 2.5[3]	♀232.3 ± 9.3	♀173.6 ± 8.7[1]<br>151.6 ± 5.8[2]<br>128.0 ± 6.3[3]		
Healthy, (mg/kg) [72]	12.4 ± 12.1	NA	156.5 ± 74.5	NA	NA	NA
Healthy, (mg/kg) [73]	8.7 ± 3.1	NA	175.1 ± 57.7	NA	NA	NA
Healthy, (µg/g) [74]	♂ 14.9 ± 0.9	NA	♂ 200.9 ± 9.7	NA	NA	NA
	♀ 15.3 ± 0.8		♀ 209.8 ± 9.5			

NA – not assessed; * – calculated from the presented data; age groups: † – 46-60, ‡ – 61-75, ¶ – 76-90; [1] – 1st myocardial infarction, [2] – 2nd, [3] – 3rd. 1 ppm = 1 µg/g = 1 mg/kg = 0.1 mg/100 g
